# Efficacy and Safety of Pleural Cryobiopsy vs. Forceps Biopsy for Evaluation of Undiagnosed Pleural Effusion: A Systematic Review and Meta-Analysis

**DOI:** 10.3389/fmed.2022.847146

**Published:** 2022-04-11

**Authors:** Mohan Giri, Haiyun Dai, Shuliang Guo, Yishi Li, Lin He, Rongjuan Zhuang

**Affiliations:** ^1^Department of Pulmonary and Critical Care Medicine, The First Affiliated Hospital of Chongqing Medical University, Chongqing, China; ^2^Department of Respiratory and Critical Care Medicine, Fuling Central Hospital, Chongqing, China

**Keywords:** pleural effusion, pleural cryobiopsy, forceps biopsy, meta-analysis, pleuroscopy

## Abstract

**Background:**

Pleural cryobiopsy is a novel technique for the diagnosis of pleural pathologies. However, the safety and feasibility of this modality compared to standard forceps for pleural biopsy has not been fully elucidated. This systematic review and meta-analysis aims to establish the efficacy and safety of cryobiopsy for evaluation of undiagnosed pleural effusion.

**Methods:**

For this systematic review and meta-analysis, we searched PubMed, Embase, Scopus, and Web of science databases up to December 16, 2021 to identify relevant articles. We included randomized controlled trials, cohort studies, retrospectives studies and case series that compared pleural cryobiopsy and forceps biopsy. A qualitative assessment was performed using the QUADAS-2 tool.

**Results:**

Of the 365 articles identified by our search, 15 studies were eligible for inclusion. The specimen sizes obtained with cryobiopsy were significantly larger compared with forceps biopsy (Standard mean difference 1.16; 95 % CI: 0.51–1.82; *P* < 0.01). Furthermore, the cryobiopsy tissue specimens were deeper (OR 2.68; 95 % CI: 1.39–5.16; *P* < 0.01) and qualitatively better with less crush artifacts (OR 0.06; 95 % CI: 0.01–0.26; *P* < 0.01). There was no significant difference in diagnostic yield (OR 1.32; 95 % CI: 0.79–2.21; *P* = 0.29) and mild to moderate bleeding events (OR 1.21; 95 % CI: 0.64–2.29; *P* = 0.57) between pleural cryobiopsy and forceps biopsy. No publication bias was observed among these studies.

**Conclusions:**

Compared to flexible forceps biopsy pleural cryobiopsy obtained larger and deeper tissue specimens with less crush artifacts but does not show superiority for diagnostic yield. Further studies are still needed to verify these findings.

## Introduction

The accurate diagnosis of pleural effusion is challenging and undiagnosed pleural effusion is frequently encountered in about 10–20 % cases, even after thoracentesis and closed pleural biopsy (1, 2). Medical thoracoscopy or video-assisted thoracoscopic surgery (VATS) performed using rigid or semi-rigid thoracoscope plays vital role for evaluating undiagnosed pleural effusion (3). Traditionally rigid pleuroscopy has been the procedure of choice as it offers larger pleural biopsy specimens with greater ease than semi-rigid pleuroscopy (4). However, in resource-limited settings rigid pleuroscopy may not be available and is more expensive than semi-rigid pleuroscopy. Although, the pleural biopsy specimens obtained during semi-rigid thoracoscopy are smaller but it is widely available and has good sensitivity (91%) and specificity (100%) in the diagnosis of exudative pleural effusion (5). Procuring adequate samples with sufficient depth from thickened or fibrosed pleura remain the most important limitation of semi-rigid pleuroscopy. Therefore, there is an immense need of alternative technology that could allow adequate biopsies of fibrotic pleura with larger specimen size to enhance the diagnostic yield. In recent years, cryotechnology has emerged as a promising tool for treating benign and malignant lung diseases (6). In addition to the therapeutic purpose cryotechnology is widely used for diagnostic purposes in interstitial lung disease, lung tumors, and in determination of lung rejection in transplant patients (7–9). Cryoprobe-based therapy is based on the Joule–Thomson effect whereby a liquefied gas exits at a high flow expands rapidly resulting in very low temperature at the tip of cryoprobe. Furthermore, the development of cryoadhesion or cryorecanalization has revolutionized the field of bronchology and introduced cryobiopsy (CB) as a promising sampling technique. The specimens obtained by cryobiopsy are larger and better-preserved with less crush artifact than traditional forceps biopsy (10, 11).

Several studies have compared the diagnostic yield, specimen size, bleeding severity, tissue depth of pleural CB and forceps biopsy (FB), but the results of these studies have been heterogeneous (12–21). Furthermore, most of these studies were conducted either with small populations or retrospectively. A recent meta-analysis by Shafiq et al. reported diagnostic yield of 96.5% for pleural cryobiopsy and 93.1% for forceps biopsy (22). However, in that meta-analysis authors pooled the results of only seven observational studies and failed to include randomized crossover study (23) that compared pleural cryobiopsy and forceps biopsy for the diagnosis of pleural effusions. In addition, they omitted pooled analysis of many efficacy (such as specimen size, biopsy death and crush artifacts) and safety endpoints (i.e., bleedings severity). Similarly, meta-analysis by Rial et al. (24) only pooled data of diagnostic yield and showed that pleural cryobiopsy was not superior to forceps biopsies. However, no meta-analysis, to date, has examined the efficacy of pleural cryobiopsy specifically for the specimen size harvested in subjects with undiagnosed pleural effusion. We therefore conducted a systemic review and meta-analysis to assess the efficacy and safety of cryobiopsy vs. forceps biopsy for evaluation of undiagnosed pleural effusion and attempted to ascertain whether there are variability in efficacy and safety endpoints with the two techniques. Our meta-analysis comparing the efficacy and safety of the pleural cryobiopsy vs. forceps biopsy is the largest to date, as we included more than twice the number of patients included in any previous meta-analysis (22, 24).

## Methods

The reporting of current meta-analysis was in accordance with the recommendations of the Preferred Reporting Items for Systematic Reviews and Meta-Analyses (PRISMA) (25).

### Search Strategy and Selection Criteria

The PubMed, Embase, Scopus, and Web of Sciences databases were searched for relevant articles. The last search was performed on December 16, 2021. The following search strategy (Cryobiopsy OR Cryoprobe biopsy OR Forceps biopsy OR pleural cryobiopsy) AND (Pleura OR Pleural effusion OR Pleural biopsy OR Pleuroscopy OR Thoracoscopy) was employed to identify all relevant studies. The full search strategies for all databases are available in Supplementary Appendix 1. We assessed all of the references of selected articles to include additional studies.

The inclusion criteria were as follows: (1) Study population: Studies in which adult patients undergoing pleural biopsy either by pleuroscopy or by thoracoscopy; (2) Comparative studies: Studies that compare cryobiopsy and forceps biopsy; (3) Outcome included overall diagnostic yield, bleeding severity, specimen size, crush artifacts, and depth of specimen. Randomized controlled trials, cohort studies, retrospectives studies and case series were included. Exclusion criteria were studies with <5 subjects, non-comparatives studies, and review articles. We performed electronic search without any time and language restrictions.

### Data Extraction

All duplicate studies were excluded by using by EndNote X 8.0 software. The two investigators (M.G. and H.Y.D.) who performed the literature search also independently extracted the data from included studies. Disagreements were resolved with a third investigator. Using a standardized data extraction form two independent reviewers abstracted the data. The extracted data included first author, year of publication, age, percent male, type of study, specimen size, diagnostic rate, bleeding severity, depth of the tissue and presence of artifacts. For continuous outcome such as specimen size, we abstracted mean and standard deviation. When only median and range were reported in studies, we calculated mean and standard deviation according to the Wan et al. (26).

### Types of Outcome Measures

The primary outcome was standardized mean difference (SMD) of sample size obtained by cryobiopsy vs. forceps biopsy. Secondary outcomes were diagnostic yield, biopsy depth, crush artifacts, and bleeding severity for these two types of biopsy methods.

### Quality Assessment

Two authors (M.G. and H.Y.D.) independently assessed the quality of individual studies using Quality Assessment of Diagnostic Accuracy Studies 2 score (QUADAS-2) tool (27). Disagreements among the reviewers were discussed and resolved during a consensus meeting. Based on the QUADAS-2 tool, each article was evaluated for risk of bias in 4 domains: (1) patient selection, (2) index test, (3) reference standard, (4) flow and timing. For each domain, the risk of bias and concerns about applicability (which also include patient selection, index test and reference standard) were analyzed and rated as low, high or unclear risk. Interrater agreement of QUADAS-2 ratings were assessed using Cohen kappa statistic.

### Statistical Analysis of Data

All statistical analysis were performed with the R Statistical Software Package 4.1.0. Odds ratio (OR) with 95 % confidence interval (CI) was calculated for dichotomous data and standard mean difference (SMD) with corresponding 95% CI for continuous data. Statistical significance was set at *P* < 0.05. I^2^ and *P*-values were calculated to assess the heterogeneity among the included studies. A *P*-value < 0.1 and I^2^ value >50% indicated substantial heterogeneity across studies. Due to the wide variation in institutional protocols for performing pleural cryobiopsy as there is no standardized methodology to perform it, we conducted all the analysis using a random-effect model. Publication bias was assessed using funnel plots and the Egger's test or the Harbord's test. Sensitivity analysis was performed to evaluate the influence of each study on the overall effect size by using the leave-one-out method (i.e., by removing one study at a time).

## Results

### Description of Included Studies

**Figure 1:**
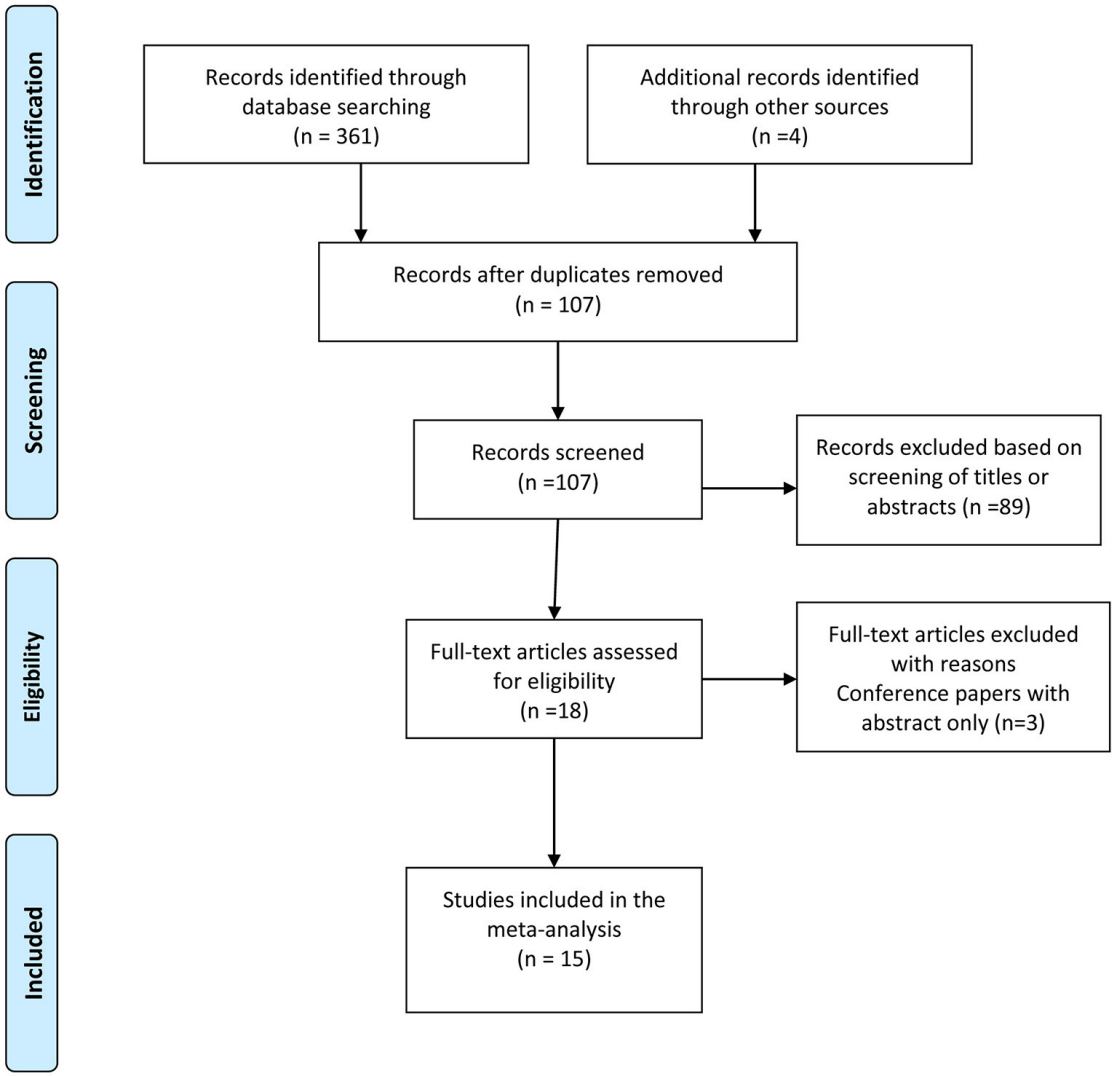
PRISMA Flow chart of study selection process.

Figure 1 shows the details of study selection process. Of the 361 records identified from literature search and four potentially eligible studies from additional source. After title and abstract screening, we assessed 18 full text articles, of which 15 were included in the meta-analysis. Three studies were excluded because they were conference article with abstract only. All studies were published after 2015. Among those 15 studies, eight prospective studies (12, 15, 17, 20, 21, 28–30), four retrospective studies (13, 18, 19, 31), two cases series (14, 16) and one randomized controlled trial (23) were analyzed, comprising 1061 biopsies (555 cryobiopsies and 506 forceps biopsies). Cryobiopsy of pleura was obtained in most patients (in 11 out of 15 studies) using cryoprobe 2.4 mm diameter. Detailed characteristics of the included studies are shown in Table 1.

**Table 1:** Characteristics of included studies.

				**Cryoprobe**	**CB diagnostic**	**FB diagnostic**
**References**	**Study type**	**Selection criteria**	**Age^**a**^**	**size (mm)**	**rate (%)**	**rate (%)**
Ahmed et al. (31)	Retrospective	Undiagnosed exudative pleural effusion	54 ± NR	3	23/30 (76.7)	23/30 (76.7)
Baess et al. (28)	Prospective	Undiagnosed exudative pleural effusion	53.6 ± 15.1	2.4	24/24 (100)	24/24 (100)
Chen et al. (12)	Prospective	Unexplained unilateral pleural effusion	64.8 (22–92)	1.9	91/92 (98.9)	84/92 (91.3)
Dhooria et al. (23)	Randomized	Undiagnosed exudative pleural effusion	53 (39–65)	2.4	39/50 (78)	38/50 (76)
	controlled
El Sayad (29)	Prospective	Undiagnosed exudative pleural effusion	CB: 55.5 ± 10.9	2.8	26/26 (100)	25/25 (100)
			FB: 52.92 ± 8.45
Ismail et al. (30)	Prospective	Undiagnosed exudative pleural effusion	62.92 ± 14.64	2.4	50/50 (100)	50/50 (100)
Lee et al. (13)	Retrospective	Undiagnosed pleural effusion	64.4 (55.4–76.4)	1.9	25/28 (89.3)	15/17 (88.2)
Maturu (14)	Case series	Undiagnosed exudative pleural effusion	50 (29-61)	2.4	6/6 (100)	3/4 (75)
Muhammad (15)	Prospective	Undiagnosed exudative pleural effusion	51.03 ± 7.518	2.4	30/30 (100)	30/30 (100)
Nakai et al. (16)	Case series	Undiagnosed pleural effusion	67.6 ± 6.15	2.4	5/5 (100)	1/5 (20)
Pathak et al. (17)	Prospective	Undiagnosed exudative pleural effusion	69 ± 11	2.4	10/10 (100)	10/10 (100)
Rozman et al. (21)	Prospective	Undiagnosed exudative pleural effusion	61 (33–83)	2.4	14/15 (93.3)	15/15 (100)
Thomas et al. (18)	Retrospective	Undiagnosed pleural effusion	72 (47–89)	2.4	20/22 (90)	20/22 (90)
Tousheed et al. (19)	Retrospective	Undiagnosed exudative pleural effusion	54.51 ± 14.99	2.4	86/87 (99)	50/52 (96.1)
Wurps et al. (20)	Prospective	Undiagnosed exudative pleural effusion	67.5 ± 13.5	2.4	73/80 (91.3)	74/80 (92.5)

*CB, cryobiopsy; FB, forceps biopsy; NR, Not reported*.

a *Values are mean ± SD or mean (range)*.

### Quality Assessment

Summary of QUADAS-2 assessments of included studies is summarized in Supplementary Figure 1. The quality of included studies was generally fair. A higher risk of bias was mainly due to inappropriate patient selection and index test, and only few studies scored high risk in quality assessment (14, 16, 17, 20, 31). In most of studies, there was low or unclear risk of bias. The Interrater agreement for quality assessment of included studies between both reviewers was very good, with Cohen kappa being 88.3 %.

### Primary Outcome

#### Specimen Size

**Figure 2:**
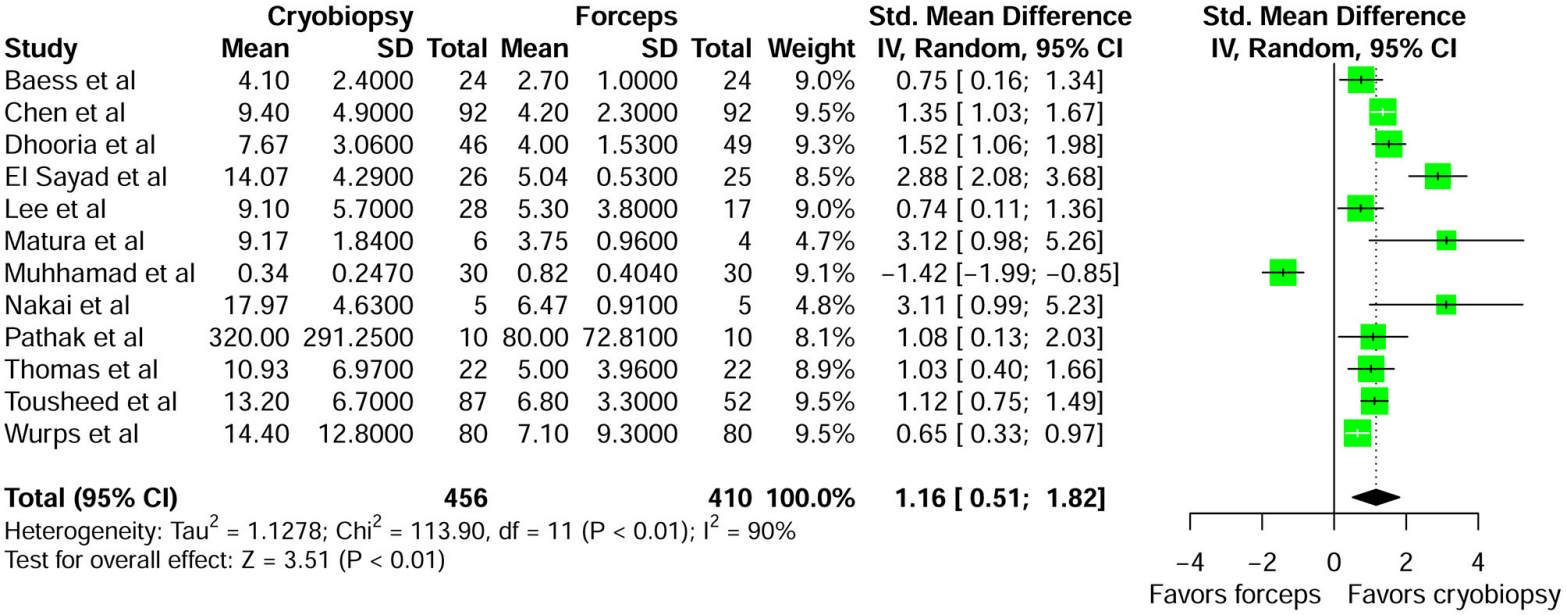
Forest plot comparing specimen size of pleural cryobiopsy vs. forceps biopsy.

A total of 12 studies were included in the pooled analysis of specimen size (Supplementary Table 1). The results of meta-analysis showed that the pooled standardized mean difference (SMD) for specimen size with cryobiopsy vs. forceps biopsy was 1.16 (95 % CI: 0.51–1.82; *P* < 0.01) (Figure 2). The above value indicated that the biopsies from cryobiopsy were significantly larger than specimens obtained by forceps biopsy. The heterogeneity was significant (I^2^ = 90%, *P* < 0.01). No evidence of publication bias was detected by the Egger's test (*P* = 0.57) and visual inspection of funnel plot (Supplementary Figure 2). The sensitivity analysis showed that no single study significantly affected the final pooled estimates of specimen size (Supplementary Figure 3).

### Secondary Outcomes

#### Diagnostic Yield

**Figure 3:**
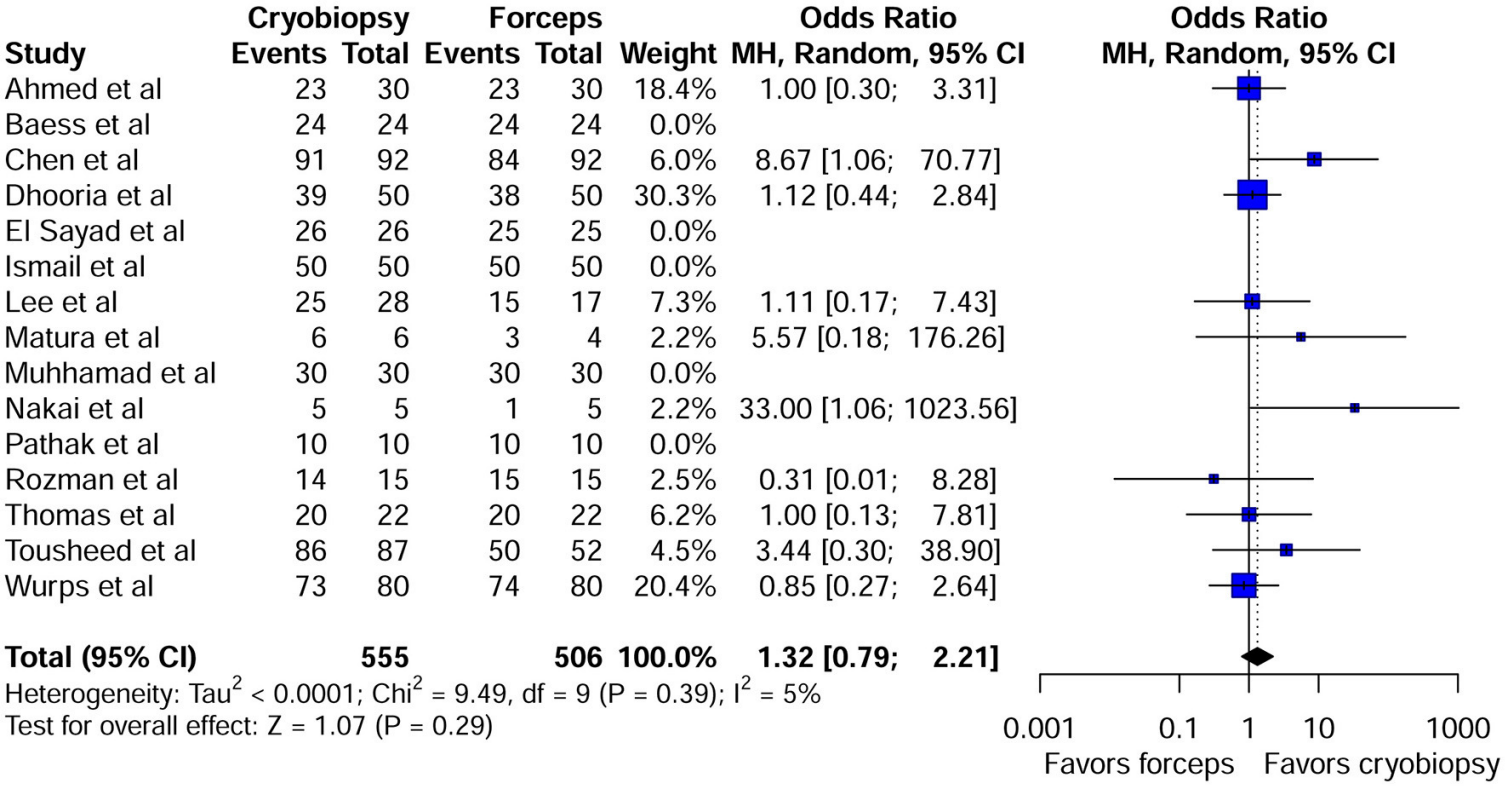
Forest plot comparing diagnostic yield of pleural cryobiopsy vs. forceps biopsy.

Overall, 15 studies compared diagnostic rate of pleural cryobiopsy with forceps biopsy. The pooled diagnostic yield of cryobiopsy was 94.1 % (522/555) and forceps biopsy was 91.3 % (462/506). Compared with forceps biopsy, cryobiopsy was not associated with a significant increase in diagnostic rate (OR 1.32; 95 % CI: 0.79–2.21; *P* = 0.29) in patients with unexplained pleural effusion (Figure 3). The heterogeneity was not significant (I^2^ = 5%; *P* = 0.39). The visual inspection of funnel plot showed roughly symmetrical distribution of studies (Supplementary Figure 4). However, the Harbord test did not show evidence of publication bias (*P* = 0.38). With respect to diagnostic rate, the direction and magnitude of the pooled ORs did not vary substantially with leave-one-out method, showing robustness of our finding (Supplementary Figure 5).

### Crush Artifacts and Biopsy Depth

**Figure 4:**
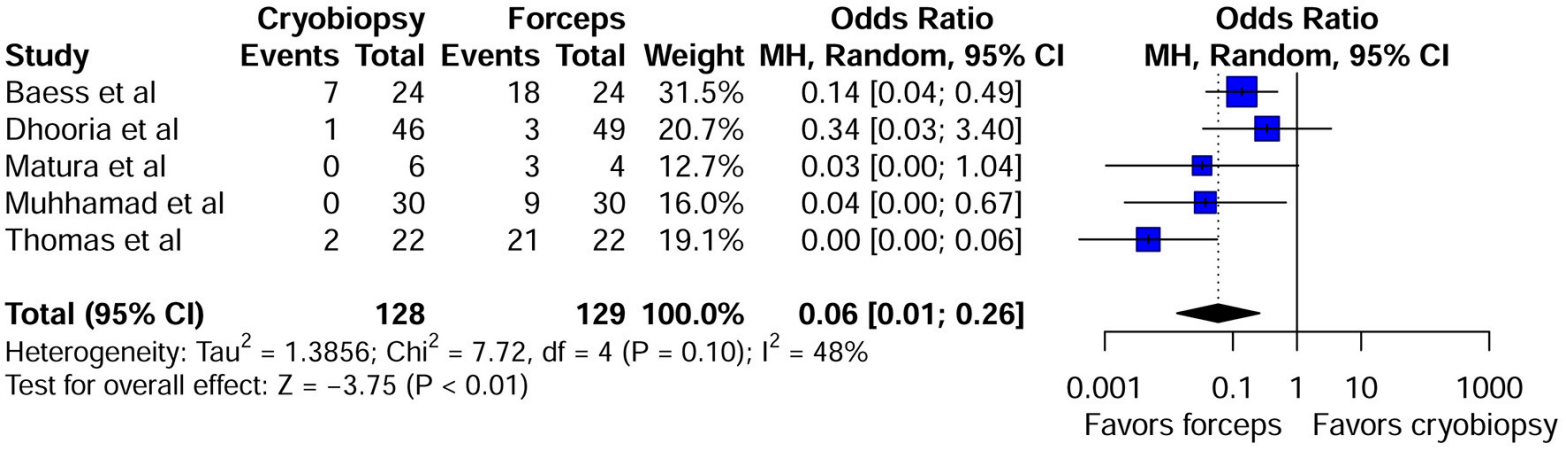
Forest plot of crush artifacts for pleural cryobiopsy vs. forceps biopsy.

Crush artifact in pleural biopsy specimens can make pathological interpretation very challenging. Five studies reported data on crush artifacts (Supplementary Table 2). Compared with forceps biopsy, cryobiopsy specimens tended to be artifacts that were less crushed and had better tissue integrity (OR 0.04; 95 % CI: 0.01–0.26; *P* < 0.01) (Figure 4). This effect size was robust in the leave-one-out sensitivity analysis (Supplementary Figure 6). Visual inspection of funnel plot (Supplementary Figure 7) and the Harbord test did not show evidence of publication bias (*P* = 0.83). Biopsy depth was reported in seven studies (Supplementary Table 3). Pleural cryobiopsy was highly successful in obtaining deeper tissue (up to the pleural fat or deeper) than that of forceps biopsy (OR 2.68; 95 % CI: 1.39–5.16; *P* < 0.01) (Supplementary Figure 8). There was moderate heterogeneity among the studies (I^2^ = 48%; *P* = 0.07). Visual inspection of the funnel plot (Supplementary Figure 9) did not show asymmetry and the Harbord test (*P* > 0.05) also revealed no significant publication bias. Sensitivity analysis using the leave-one-out approach showed that our result was robust (Supplementary Figure 10).

### Bleeding Severity

Ten studies evaluated bleeding severity (Table 2) of which six studies contributed to the pooled analysis of bleeding event. Mild to moderate bleeding events were not significantly different between cryobiopsy and forceps biopsy group (OR 1.21; 95 % CI: 0.64–2.29; *P* = 0.57) (Supplementary Figure 11). There was no evidence of heterogeneity among pooled studies (I^2^ = 0%, *P* = 0.92). Funnel plot showed relatively symmetrical plot (Supplementary Figure 12) and no publication bias was detected by the Harbord test (*P* > 0.05). Furthermore, sensitivity analysis indicated that the results were robust (Supplementary Figure 13).

**Table 2:** Qualitative analysis of bleeding severity.

		**Number of patients**	**Number of patients**
**Author**	**Definition of bleeding severity**	**in CB, *n* (%)**	**in FB, *n* (%)**
Ahmed et al.	Mild bleeding	0/30 (0)	1/30 (3.33)
Baess et al.	NR	NR	NR
Chen et al.	Nil: slight, self-limited	84/92 (91.3)	86/92 (93.5)
	Mild: requiring vasoactive drug (adrenaline) injection	8/92 (8.7)	6/92 (6.5)
	Moderate to severe: requiring electrocautery or APC intervention	0/92 (0)	0/92 (0)
Dhooria et al.	Minimal:self-limited ooze	46/46 (100)	49/49 (100)
	Mild: requiring prolonged suctioning	0/46 (0)	0/49 (0)
	Major: requiring blood transfusion, causing hemodynamic instability or ICU admission	0/46 (0)	0/49 (0)
Ismail et al.	NR	NR	NR
El Sayad et al.	Self-limited	16/26 (61.5)	15/25 (60)
	Mild bleeding	10/26 (38.5)	10/25 (40)
	Moderate to severe	0/26 (0)	0/25 (0)
Lee et al.	No bleeding	25/28 (89.3)	16/17 (94.1)
	Mild: self-limiting	3/28 (10.7)	1/17 (5.9)
	Moderate: electrocautery application for hemostasis	0 (0)	0 (0)
	Severe: intravenous resuscitation, blood transfusion, and surgical or radiological interventions required	0 (0)	0 (0)
Matura et al.	NR	0/6	NR
Muhhamad et al.	NR	NR	NR
Nakai et al.	Mild bleeding	1/5 (20)	0/5 (0)
Pathak et al.	NR	NR	NR
Rozman et al.	Slight: self-limited	42/42 (100)	NR
	Moderate: electrocautery intervention	0/42 (0)	NR
	Severe: interruption of the procedure, chest tube drainage and iv resuscitation	NR	NR
Thomas et al.	Nil bleeding	17/22 (77.3)	18/22 (81.8)
	Mild: self-limiting	5/22 (22.7)	4/22 (18.2)
	Moderate: electrocautery application for hemostasis	0/22 (0)	0/22 (0)
	Severe: intravenous resuscitation, blood transfusion and/or surgical or radiological interventions	0/22 (0)	0/22 (0)
Tousheed et al.	Minimal bleeding	87/87 (100)	NR
Wurps et al.	Moderate to severe bleeding	0/6 (0)	0/6 (0)

*CB, cryobiopsy; FB, forceps biopsy; APC, argon plasma coagulation; NR, Not reported; ICU, intensive care unit*.

## Discussion

Pleural biopsy is indicated for unexplained pleural effusion that remains undiagnosed even after radiological imaging and pleural fluid analysis. Pleural cryobiopsy is evolving as the new technique for diagnosing exudative pleural effusion, when the diagnosis has remained elusive despite one or two thoracentesis. In fact, only limited studies have been conducted that compared pleural cryobiopsy and forceps based pleural biopsy. In the present meta-analysis of 15 articles involving 1061 biopsies with undiagnosed pleural effusion who underwent pleural cryobiopsy and forceps biopsy, we found that efficacy/safety outcome of specimen size, biopsy depth and crush artifacts differ significantly between cryobiopsy and forceps biopsy. However, diagnostic yield and bleeding events were similar between the groups.

This meta-analysis showed that the specimens obtained using cryobiopsy were significantly larger in size than the forceps biopsy specimens (SMD = 1.16; 95% CI: 0.51–1.82; *P* < 0.01). Previous pooled analysis of seven studies evaluating cryobiopsy and forceps biopsy also documented that biopsy size harvested by cryobiopsy were significantly larger than forceps biopsy (SMD = 0.867; 95% CI: 0.427–1.308; *P* < 0.001) (32). However, this systematic review and meta-analysis was only published as conference abstract. Conference abstracts often lack rigorous peer review, conclusions drawn from the results presented in such abstract may be biased or imprecise. In our study larger samples with sufficient extrapleural fat tissue or pleural tissue might have helped in a confident histological diagnosis of the pleural effusion in patients with thickened or fibrosed pleura. Cryobiopsy is a promising technique because both biopsy size and quality contribute to diagnostic yield. In a recent meta-analysis, Shafiq et al. (22) demonstrated that larger pleura samples were obtained through cryobiopsy than through forceps biopsy. However, they just performed qualitative analysis of specimen size and failed to perform the pooled analysis. Similarly, another meta-analysis has focused exclusively on diagnostic yield and omitted pooled analysis of other efficacy and safety end points (24). Cryobiopsy is performed using several different variations of technique across centers, this variability in institutional protocols for performing the procedure might be the reason for significant heterogeneity for biopsy size across included studies.

Our systematic review and meta-analysis revealed that the diagnostic yield of pleural cryobiopsy is comparable to that of traditional pleural biopsy using flexible forceps for undiagnosed pleural effusion (OR 1.32; 95 % CI: 0.79–2.21; *P* = 0.29). In a recent study, Chen et al. (12) showed that cryobiopsy during semi-rigid pleuroscopy was a safe technique with a higher diagnostic yield than forceps biopsy for the diagnosis of exudative pleural effusion (EPE). Similarly, Touseed et al. (19) revealed that diagnostic yield was 99% with cryobiopsy and 96% with forceps biopsy, however there was no significant difference in the diagnostic yield between these two techniques. The first randomized trial that has compared the yield of the two techniques found that diagnostic rate of pleural effusion with cryobiopsy was higher than forceps biopsy (CB: 78.0% Vs FFB 76 %) even though there was no statistically significant difference (23). On the other hand, in patients undergoing pleural biopsy using flexible forceps, followed by a flexible cryoprobe introduced through the pleuroscope, Thomas et al. (18) found that diagnostic yield achieved with cryobiopsies was similar to the yield of forceps biopsy. However, there was no significant improvement in diagnostic yield by combining FB with the CB in this small cohort. Similarly, a recent meta-analysis by Shafiq et al. (22) demonstrated that pleural cryobiopsy is a safe method with similar diagnostic value, comparable to flexible forceps biopsy. However, they just included seven observational studies in their meta-analysis, which was less than the number of our included studies. Furthermore, we included the first ever randomized crossover study that compared pleural cryobiopsy and flexible forceps biopsy in subjects undergoing medical thoracoscopy for the diagnosis of pleural effusions (23). Despite its remarkable ability to harvest significantly deeper and larger specimens with less crush artifact, pleural cryobiopsy does not show superiority for diagnostic yield over forceps biopsy. Given that both techniques produced more than a 90% diagnostic rate, larger numbers of cases should be evaluated in future studies to find a statistically significant difference. Importantly, cryobiopsies will produce better results in cases with thickened and sclerotic pleura (where a forceps biopsy can be difficult), but forceps biopsies are adequate in the vast majority of cases. Additionally, in areas with a higher prevalence of asbestos-related pleural disease, cryobiopsy may play an important role in achieving a diagnosis. Although a rigid thoracoscope can obtain larger biopsy specimens from thickened pleura, its maneuverability in the pleural space is limited.

The definition of bleeding severity varied across studies. In most of the studies, slight or self-limited bleeding was reported which was similar between cryobiopsy and forceps biopsy groups (12, 13, 18, 21, 23). Our pooled analysis revealed that there were no significant differences in the mild to moderate bleeding events between the cryobiopsy and forceps biopsy groups. No severe bleeding was reported that required blood transfusion, causing hemodynamic instability or ICU admission in the individual studies included in this meta-analysis. More RCTs assessing bleeding severity in standardized way are required to draw further conclusion regarding the safety of these biopsy methods. Histopathological diagnosis of malignant mesothelioma is particularly challenging due to the presence of diffusely thickened or fibrotic pleura (33). The role of pleural cryobiopsy is paramount in this regard as the higher number of deep biopsies containing fatty tissue should enable to detect mesothelioma more accurately compared to forceps biopsy. Pooled analysis of biopsy depth in our meta-analysis also revealed that the cryobiopsy was able to obtain biopsies containing fatty tissue or deeper layer than forceps biopsy. The study performed by Shafiq et al. (22) incorporated a similar result but they did not perform pooled analysis of biopsy depth. The presence of artifacts in histological sections obtained by flexible forceps biopsy is a very common finding and represents a potentially major pitfall for the pathological diagnosis of pleural disease (34, 35). Our pooled result showed that in comparison with forceps biopsy, crush artifacts were minimal with cryobiopsy. In line with our study Shafiq et al. (22) also reported fewer instances of crush artifacts with cryobiopsy but they just performed qualitative analysis regarding artifacts. This systematic review and meta-analysis has several limitations. First, the small number of studies identified, the studies were mostly small in sample size and retrospective in design. Second, study by Nakai et al. (16) included five patients with pleural effusion, and in this study they investigated the utility of cryoprobe and conventional biopsy in the diagnosis of malignant pleural mesothelioma, so its representativeness for undiagnosed pleural effusion may be open to doubt. Third, there was pronounced variation in institutional protocols for performing pleural cryobiopsy as there is no standardized methodology to perform it. Fourth, operator's skills in performing biopsy procedure was not reported by majority of the studies. Fifth, the risk of bleeding is not robustly reported in many published studies and the definition of bleeding with cryobiopsy and forceps biopsy was not uniform. Well designed, larger multi-center randomized trials and prospective studies are warranted to provide more evidence for efficacy and safety of pleural cryobiopsy.

## Conclusions

In conclusion, despite the limitations noted, compared with forceps biopsy, cryobiopsy is relatively safe procedure with larger artifact-free specimen but does not offer high diagnostic yield. However, no meaningful conclusion can be drawn regarding severe bleeding events. Direct comparison of cryobiopsy and forceps biopsy through multi-center randomized, controlled trial would be valuable to verify our findings.

## Data Availability Statement

The original contributions presented in the study are included in the article/Supplementary Material, further inquiries can be directed to the corresponding author.

## Author Contributions

MG, HD, and SG: conceptualization and project administration. MG, SG, HD, and YL: data curation and review and editing. MG, HD, YL, SG, and LH: formal analysis. MG, HD, and RZ: investigation. SG and MG: supervision. SG, MG, RZ, LH, and YL: validation. MG, YL, RZ, and SG: visualization. MG, LH, RZ, and SG: writing. All authors contributed to the article and approved the submitted version.

## Funding

This study was supported by Research projects on key disease prevention and control technologies in Chongqing (Project number: 2019ZX002) and Chongqing Pulmonary Nodule office.

## Conflict of Interest

The authors declare that the research was conducted in the absence of any commercial or financial relationships that could be construed as a potential conflict of interest.

## Publisher's Note

All claims expressed in this article are solely those of the authors and do not necessarily represent those of their affiliated organizations, or those of the publisher, the editors and the reviewers. Any product that may be evaluated in this article, or claim that may be made by its manufacturer, is not guaranteed or endorsed by the publisher.
