# Supplementary material for: Efficacy and Safety of Pleural Cryobiopsy vs. Forceps Biopsy for Evaluation of Undiagnosed Pleural Effusion: A Systematic Review and Meta-Analysis
Source: Front Med (Lausanne). 2022 Apr 11;9:847146. doi: 10.3389/fmed.2022.847146 (PMC9035697; doi:10.3389/fmed.2022.847146)
Supplement: Supplementary file 1 [file Data_Sheet_1.pdf]

Supplementary Material

Contents

Supplementary Appendix 1. Full search terms used in the literature search of each database.

Supplementary Figure 1. Quality assessments of included studies using the QUADAS-2 tool.

Supplementary Figure 2. Funnel plots to detect publication bias for studies comparing specimen size.

Supplementary Figure 3. Leave-one-out sensitivity analysis of specimen size between cryobiopsy and forceps biopsy.

Supplementary Figure 4. Funnel plot to detect publication bias for studies comparing diagnostic yield between cryobiopsy and forceps biopsy.

Supplementary Figure 5. Forest plot of leave-one-out meta-analysis of the diagnostic yield.

Supplementary Figure 6. Forest plot of the leave-one-out meta-analysis of crush artifacts.

Supplementary Figure 7. Funnel plot to detect publication bias for studies comparing crush artifacts between cryobiopsy and forceps biopsy.

Supplementary Figure 8. Forest plot of specimen depth of pleural cryobiopsy versus forceps biopsy.

Supplementary Figure 9. Funnel plot to detect publication bias for studies comparing specimen depth between cryobiopsy and forceps biopsy.

Supplementary Figure 10. Forest plot of the leave-one-out meta-analysis of specimen depth.

Supplementary Figure 11. Forest plot of mild to moderate bleeding events occurred during pleural cryobiopsy versus forceps biopsy.

Supplementary Figure 12. Funnel plot of studies comparing cryobiopsy versus forceps biopsy for mild to moderate bleeding events.

Supplementary Figure 13. Forest plot of the leave-one-out meta-analysis of mild to moderate bleeding events.

Supplementary Table 1. Average specimen size obtained by pleural cryobiopsy and forceps biopsy.

Supplementary Table 2. Artefacts reported in included studies comparing pleural cryobiopsy versus forceps biopsy.

Supplementary Table 3. Qualitative analysis of depth of biopsy between pleural cryobiopsy and forceps biopsy.

Supplementary Appendix 1- Full search terms used in the literature search of each database.

1. PubMed

Search ID	*Search Terms*
#1	("Cryobiopsy" OR "Cryoprobe biopsy" OR "Forceps biopsy" OR "pleural cryobiopsy")
#2	(" Pleura*" OR " Pleural effusion " OR " pleural biopsy " OR "pleuroscopy" OR "thoracoscopy")
#3	#1 AND #2 (Results retrieved n= 68)

2. Embase

Search History	*Searches*
1	((('cryobiopsy'/exp OR cryobiopsy OR 'cryoprobe'/exp OR cryoprobe) AND ('biopsy'/exp OR biopsy) OR 'forceps'/exp OR forceps) AND ('biopsy'/exp OR biopsy) OR pleural) AND ('cryobiopsy'/exp OR cryobiopsy)
2	((pleura* OR pleural) AND ('effusion'/exp OR effusion) OR pleural) AND ('biopsy'/exp OR biopsy) OR 'pleuroscopy'/exp OR pleuroscopy OR 'thoracoscopy'/exp OR thoracoscopy
3	#1 AND #2 (Results retrieved n= 93)

3. Scopus

TITLE-ABS-KEY (("Cryobiopsy" OR " Cryoprobe biopsy " OR "Forceps biopsy" OR "pleural cryobiopsy") AND (" Pleura*" OR " Pleural effusion " OR " pleural biopsy " OR "pleuroscopy" OR "thoracoscopy")) (Results retrieved n= 106)

4. Web of Science

Search History	*Searches*

#1	TS=("Cryobiopsy" OR " Cryoprobe biopsy " OR “Forceps biopsy” OR “pleural cryobiopsy”)
#2	TS=(" Pleura*" OR " Pleural effusion " OR " pleural biopsy " OR "pleuroscopy" OR “thoracoscopy”)
#3	#1 AND #2 (Results retrieved n= 94)

Supplementary Figure 1. Quality assessments of included studies using the QUADAS-2 tool. A) Risk of bias graph: Review authors' judgments about each risk of bias item presented as percentages across all included randomized controlled studies. **B**) Risk of bias summary: Review authors' judgments for each risk of bias item for the included studies.

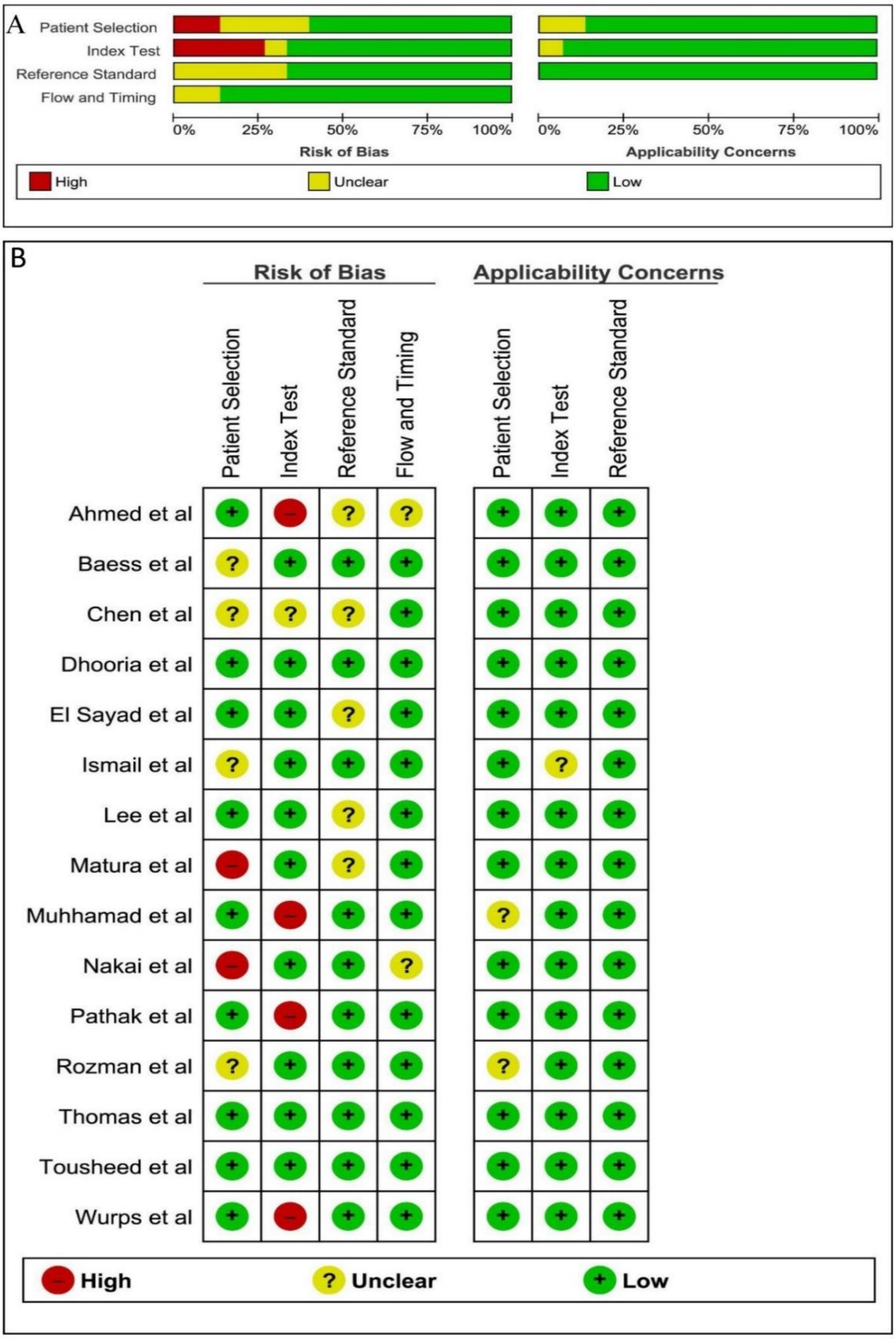

Supplementary Figure 2. Funnel plot to detect publication bias for studies comparing specimen size.

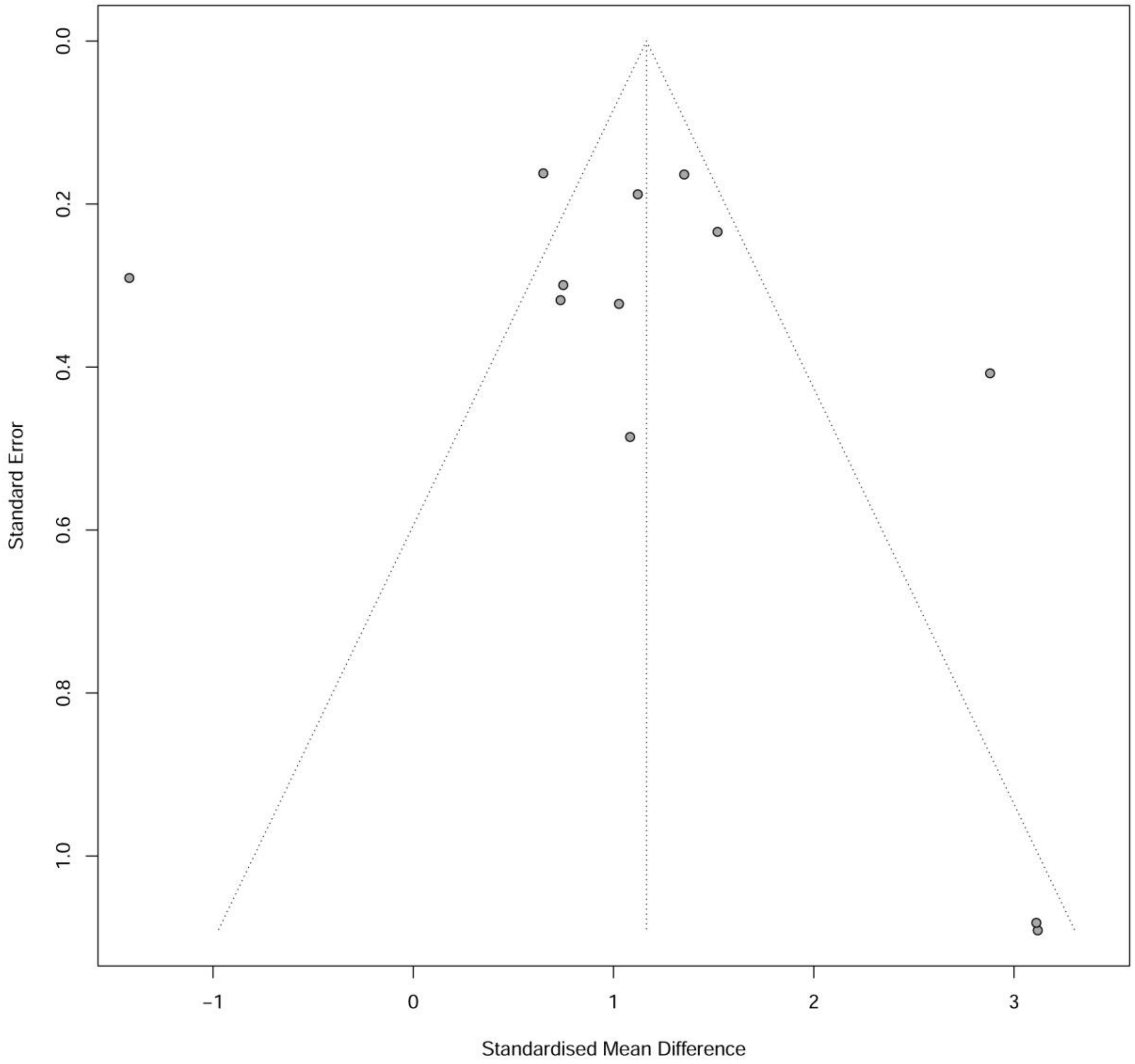

Supplementary Figure 3. Leave-one-out sensitivity analysis of specimen size between cryobiopsy and forceps biopsy

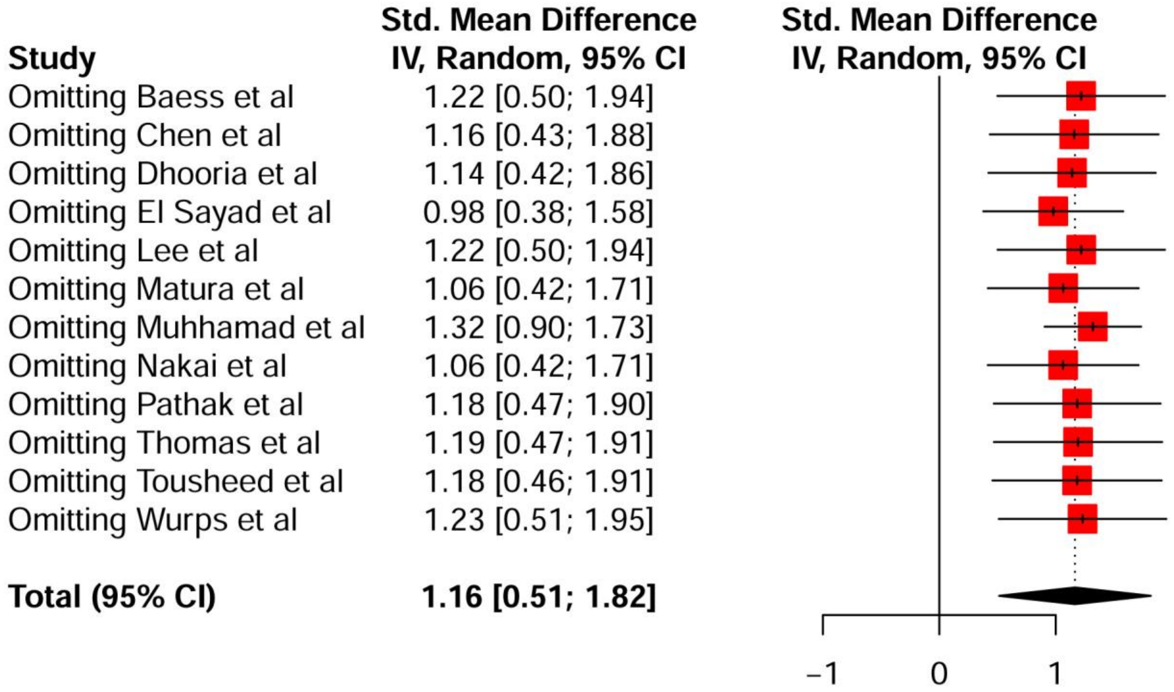

Supplementary Figure 4. Funnel plot to detect publication bias for studies comparing diagnostic yield between cryobiopsy and forceps biopsy

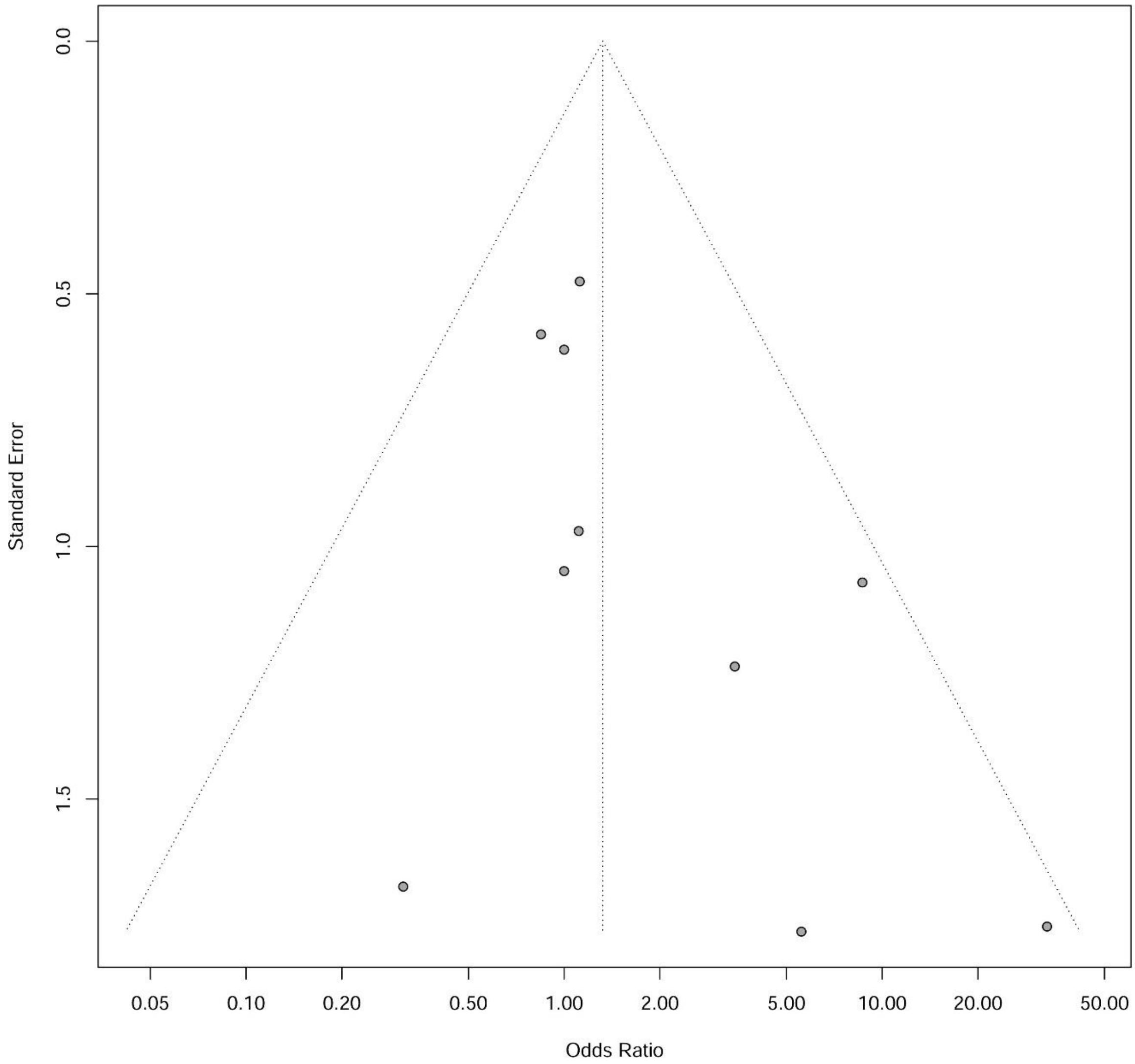

Supplementary Figure 5. Forest plot of leave-one-out meta-analysis of the diagnostic yield

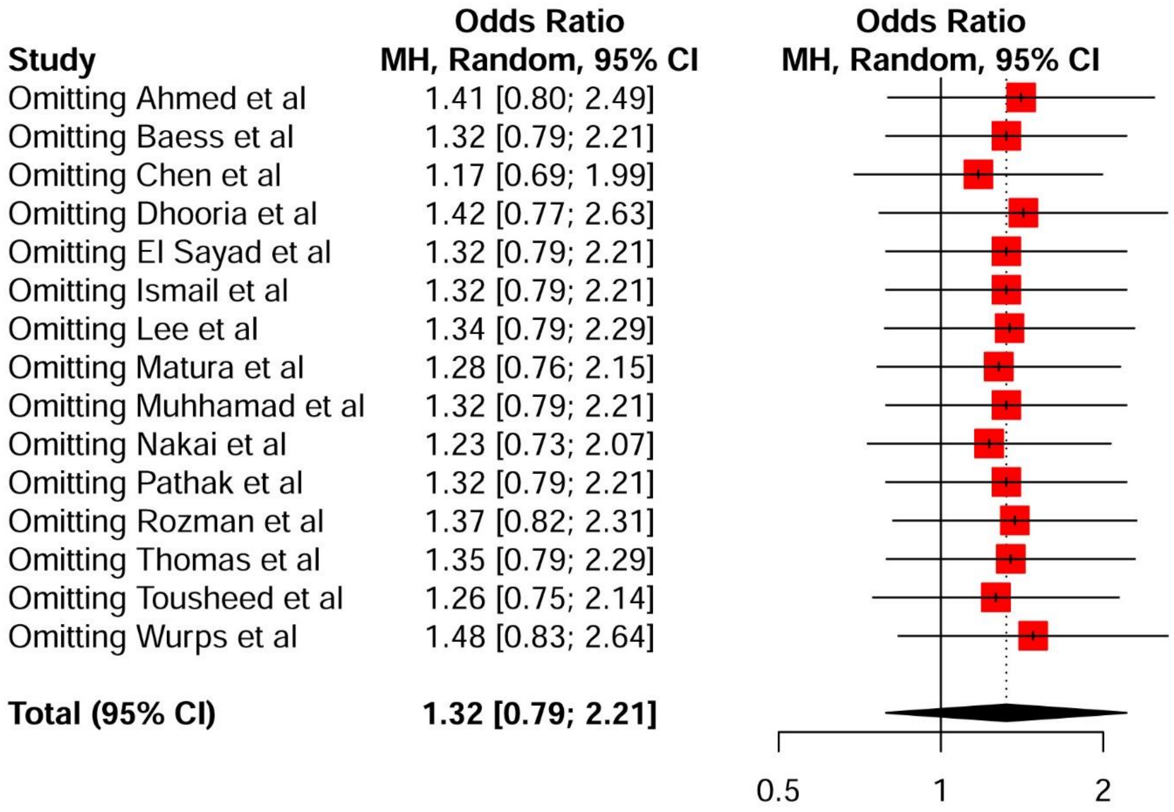

Supplementary Figure 6. Forest plot of the leave-one-out meta-analysis of crush artifacts

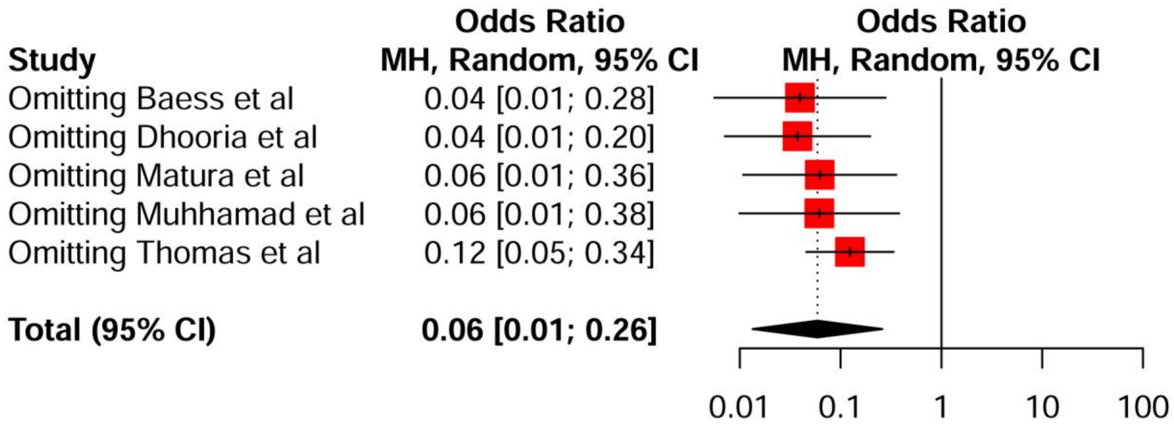

Supplementary Figure 7. Funnel plot to detect publication bias for studies comparing crush artifacts between cryobiopsy and forceps biopsy

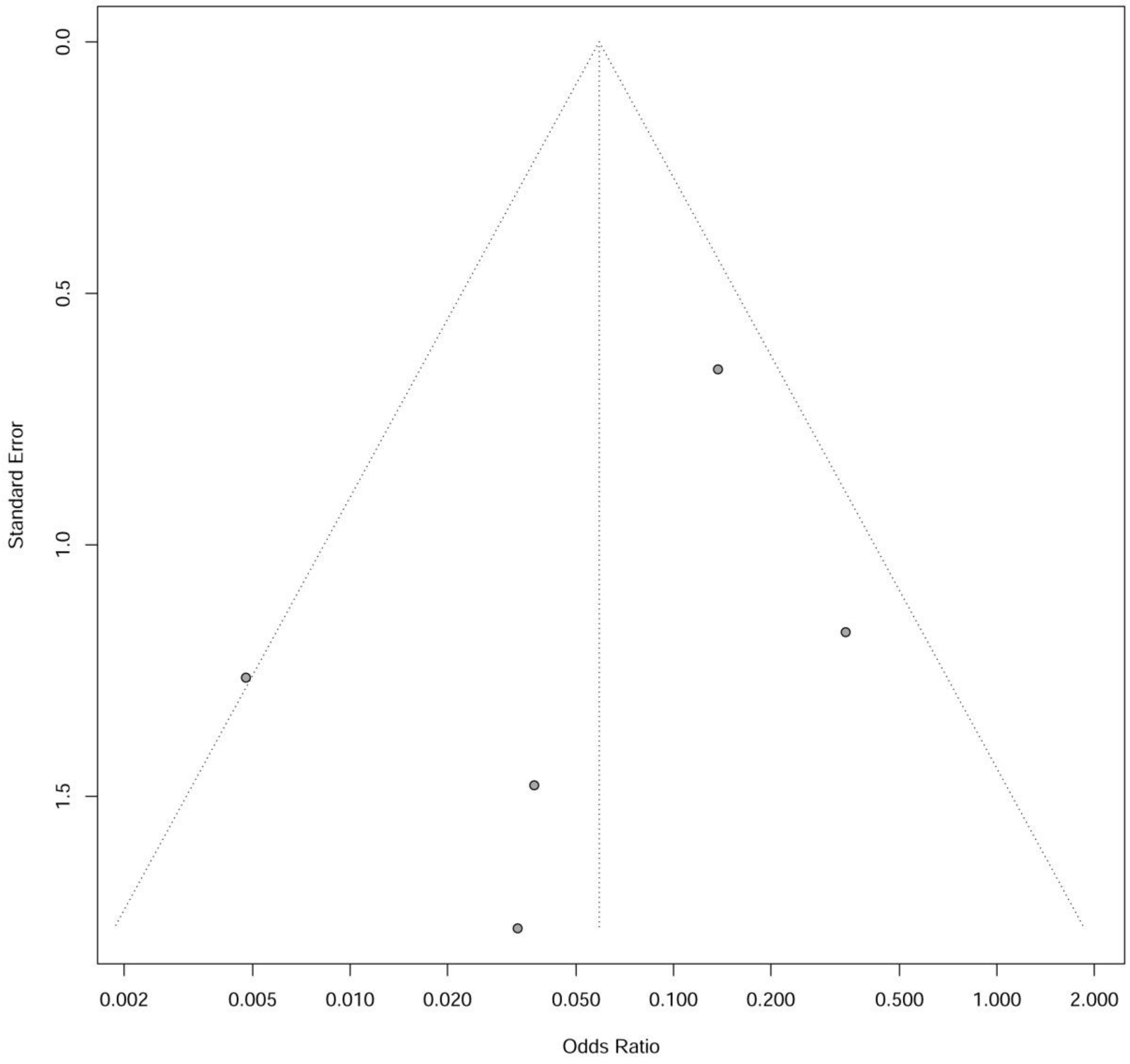

Supplementary Figure 8. Forest plot of specimen depth of pleural cryobiopsy versus forceps biopsy

	Cryobiopsy		Forceps			Odds Ratio
**Study**	**Events**	**Total**	**Events**	**Total**	**Weight**	**MH, Random, 95% CI**
Baess et al	8	24	8	24	15.7%	1.00 [0.30; 3.32]
Dhooria et al	30	46	20	49	21.6%	2.72 [1.18; 6.25]
Matura et al	4	6	0	4	3.5%	16.20 [0.59; 441.68]
Muhhamad et al	21	30	12	30	17.6%	3.50 [1.20; 10.20]
Nakai et al	2	5	1	5	4.6%	2.67 [0.16; 45.14]
Thomas et al	14	22	2	22	10.3%	17.50 [3.22; 95.16]
Wurps et al	49	99	41	104	26.7%	1.51 [0.86; 2.63]
**Total (95% CI)**		**232**		**238**	**100.0%**	**2.68 [1.39; 5.16]**

Heterogeneity: $Tau^2 = 0.3407$; $Chi^2 = 11.58$, df = 6 (P = 0.07); $I^2 = 48\%$
Test for overall effect: Z = 2.94 (P < 0.01)

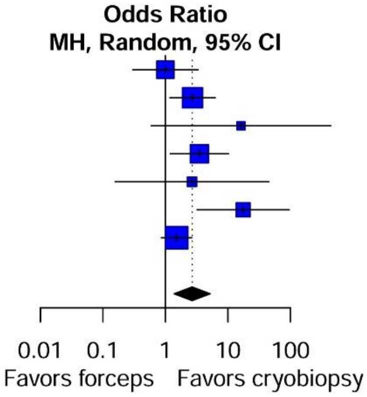

Supplementary Figure 9. Funnel plot to detect publication bias for studies comparing specimen depth between cryobiopsy and forceps biopsy

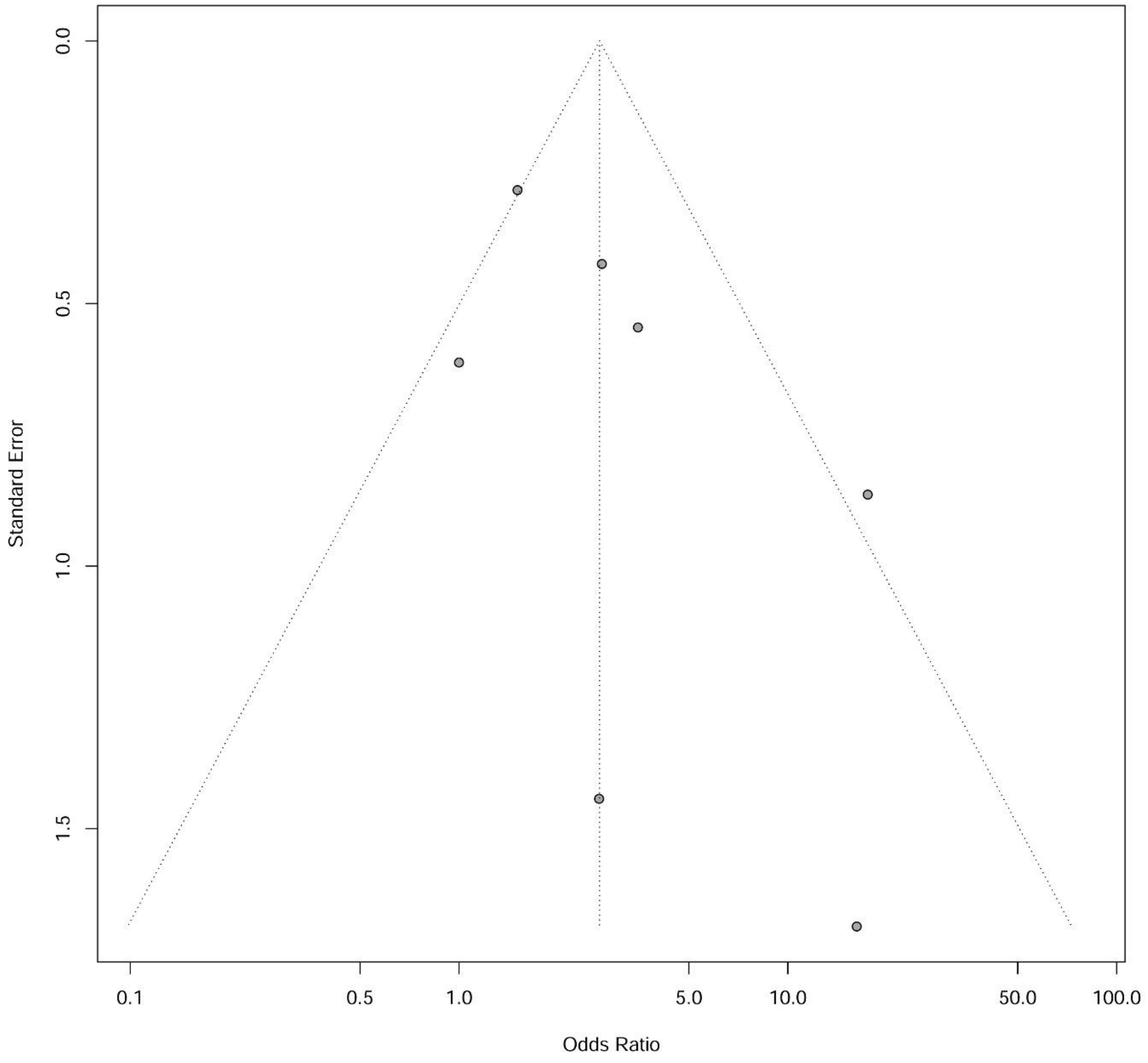

Supplementary Figure 10. Forest plot of the leave-one-out meta-analysis of specimen depth

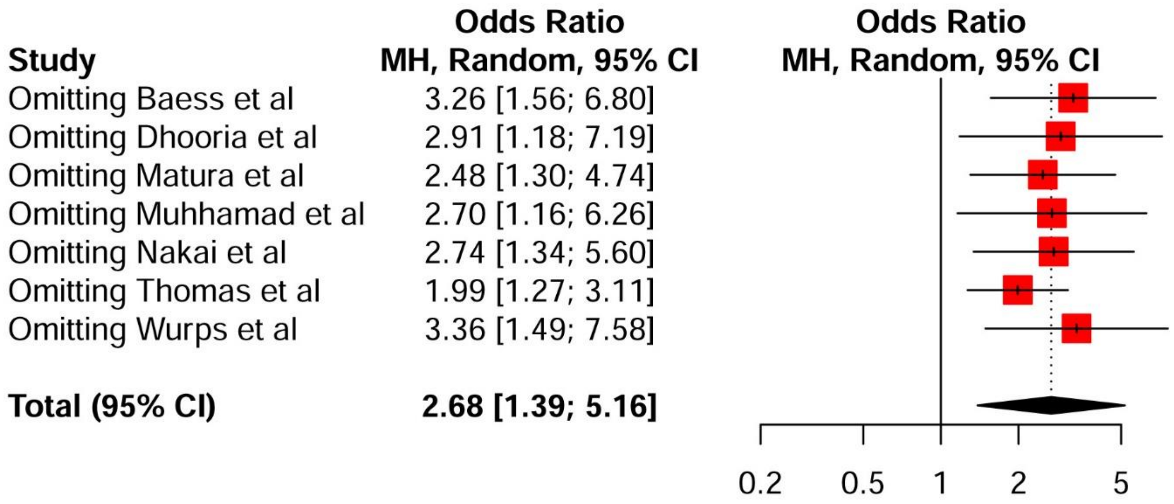

Supplementary Figure 11. Forest plot of mild to moderate bleeding events occurred during pleural cryobiopsy versus forceps biopsy

Study	Cryobiopsy Events	Cryobiopsy Total	Forceps Events	Forceps Total	Weight	Odds Ratio MH, Random, 95% CI
Ahmed et al	0	30	1	30	3.9%	0.32 [0.01; 8.24]
Chen et al	8	92	6	92	33.9%	1.37 [0.45; 4.10]
Dhooria et al	0	46	0	49	0.0%	
El Sayad et al	10	26	10	25	32.4%	0.94 [0.30; 2.89]
Lee et al	3	28	1	17	7.4%	1.92 [0.18; 20.10]
Nakai et al	1	5	0	5	3.5%	3.67 [0.12; 113.73]
Thomas et al	5	22	4	22	18.9%	1.32 [0.30; 5.77]
Wurps et al	0	6	0	6	0.0%	
**Total (95% CI)**		**255**		**246**	**100.0%**	**1.21 [0.64; 2.29]**

Heterogeneity: $Tau^2 = 0$; $Chi^2 = 1.45$, df = 5 (P = 0.92); $I^2 = 0\%$
Test for overall effect: Z = 0.57 (P = 0.57)

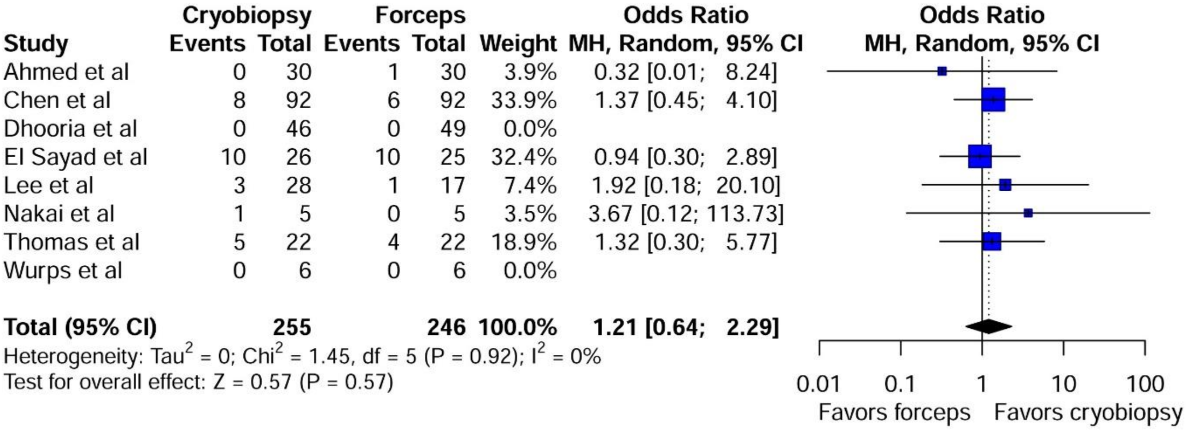

Supplementary Figure 12. Funnel plot of studies comparing cryobiopsy versus forceps biopsy for mild to moderate bleeding events

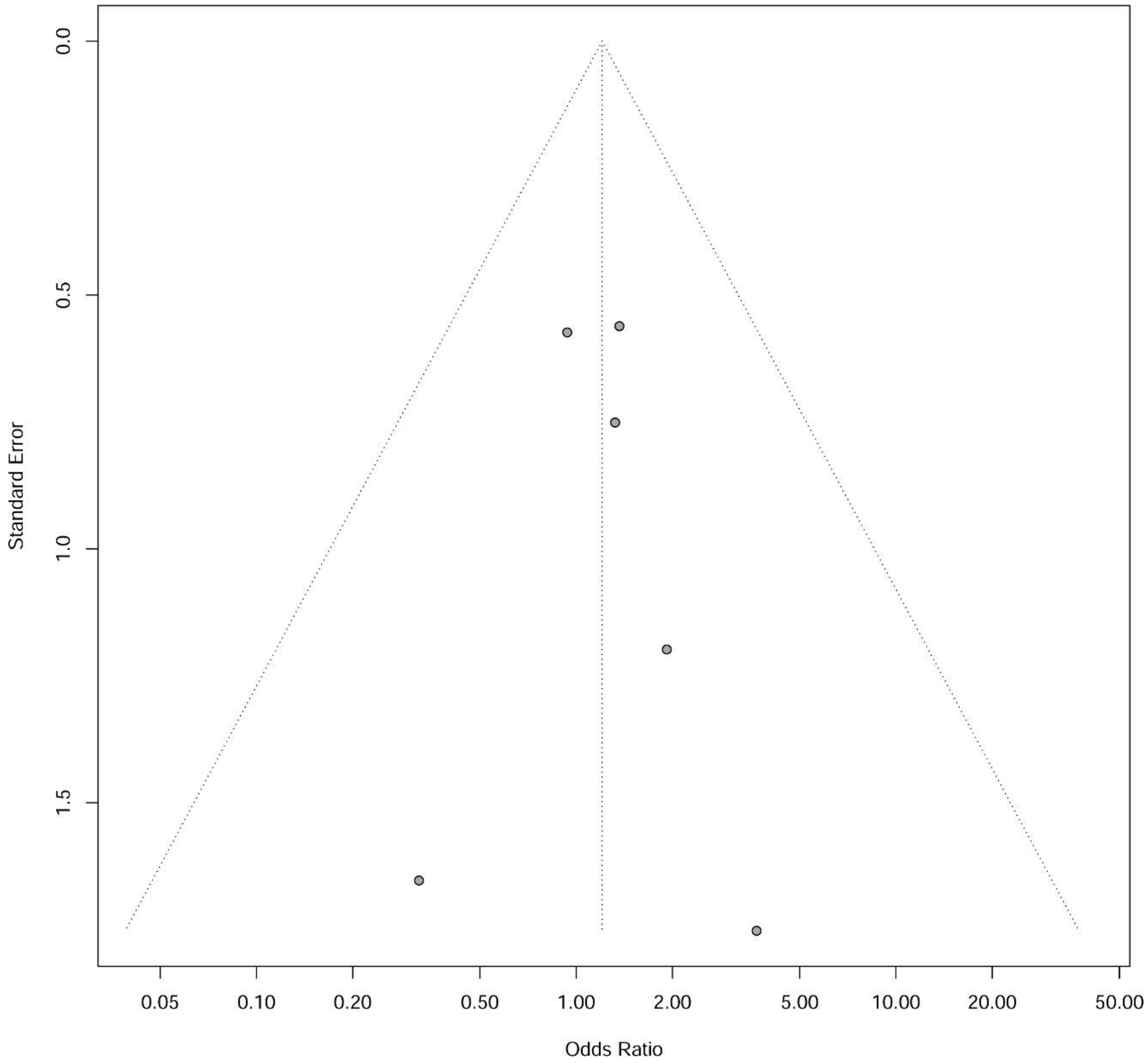

Supplementary Figure 13. Forest plot of the leave-one-out meta-analysis of mild to moderate bleeding events

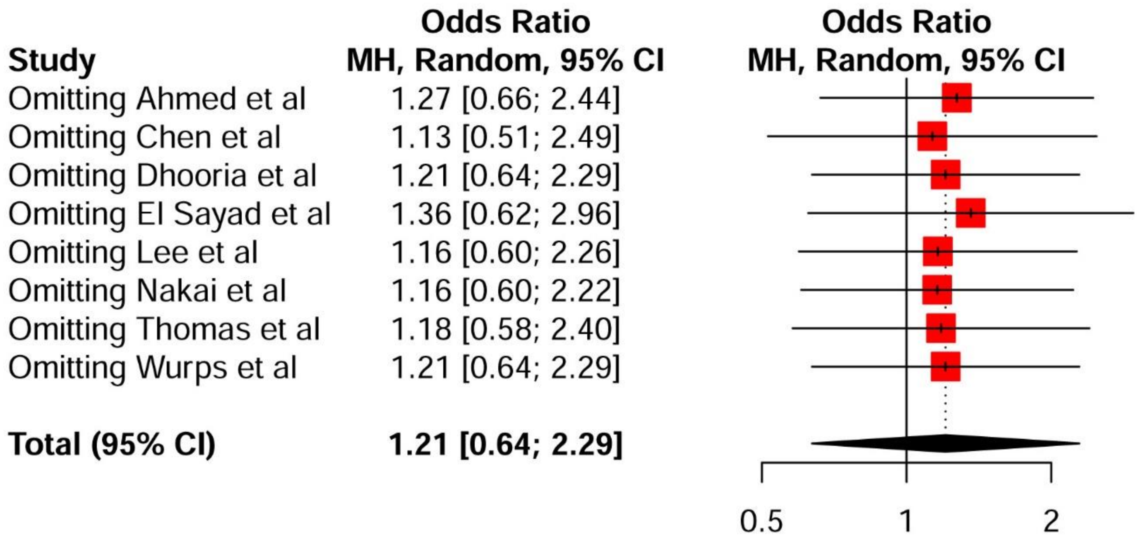

Supplementary Table 1. Average specimen size obtained by pleural cryobiopsy and forceps biopsy

Study (year)	Average biopsy size cryobiopsy	Average biopsy size forceps biopsy
Ahmed et al	NR	NR
Baess et al	4.1 ± 2.4 cm^2 (Surface area ± SD	2.7 ± 1 cm^2 (Surface area ± SD)
Chen et al[12]	9.4mm ± 4.9 mm (mean size ± SD)	4.2mm ± 2.3 mm (mean size ± SD)
Dhooria et al[23]	7 mm(6–10) (median size, range)	4 (3–5) (median size, range)
El Sayad et al	14.07 ± 4.29 (mean size ± SD)	5.04 ± 0.53 (mean size ± SD)
Ismail et al	NR	NR
Lee et al[13]	9.1 ± 5.7mm (mean size ± SD)<br>56.0 ± 61.3 mm^2 (mean area ± SD)<br>232.1 ± 411.4 mm^3 (volume ± SD)	5.3 ± 3.8 (mean size ± SD)<br>10.2 ± 14.3 mm^2 (mean area ± SD)<br>13.4 ± 22.6 mm^3 (volume ± SD)
Matura et al[14]	9.17 ± 1.84 mm (mean size ± SD)	3.75 ± 0.96 mm (mean size ± SD)
Muhhamad et al[15]	0.338±0.247 cm^2 (Surface area ± SD)	0.819±0.404 cm^2 (Surface area ± SD)
Nakai et al[16]	18.9 (15.2–19.8) mm^2 (cross-sectional area ; median, range)	6.7 (5.9–6.8) mm^2 (cross-sectional area ; median, range)
Pathak et al[17]	320 mm^3 mean tissue volume	80 mm^3 mean tissue volume
Rozman et al[21]	NR	NR
Thomas et al[18]	10 (7-15.8) mm (median size, range)	4 (3-8) mm (median size, range)
Tousheed et al[19]	13.2± 6.7 mm (mean size ± SD)	6.8± 3.3 mm (mean size ± SD)
Wurps et al[20]	14.4 ± 12.8 mm^2 (mean area ± SD)	7.1 ± 9.3 mm^2 (mean area ± SD)

NR= Not reported

Supplementary Table 2. Artefacts reported in included studies comparing pleural cryobiopsy versus forceps biopsy

Author	Occurrence of artefacts	Number of patients in CB, n (%)	Number of patients in FB, n (%)
Baess et al	Crushed cells negative	17/24 (70.8)	6/24 (25)
	Crushed cells <5%	6/24 (25)	14/24 (58.3)
	Crushed cells 5-10%	1/24 (4.2)	0/24 (0)
	Crushed cells 10-25%	0/24 (0)	4/24 (16.7)
Dooria et al	None	45/46 (97.8)	46/49 (93.9)
	Crush	1/46 (2.2)	3/49 (6.1)
	Freeze	0/46 (0)	0/49 (0)
Matura et al	Crush artefacts	0/6 (0)	3/4 (75)
Muhhamad et al	Crushed cells	0/30 (0)	9/30 (30)
	No crushed cells	30/30 (100)	21/30 (70)
Thomas et al	Crush artefacts nil	20/22 (91)	1/22 (4.5)
	Crush artefacts mild	1/22 (4.5)	19/22 (86.4)
	Crush artefacts moderate	1/22 (4.5)	2/22 (9.1)
	Crush artefacts severe	0/22 (0)	0/22 (0)

Supplementary Table 3. Qualitative analysis of depth of biopsy between pleural cryobiopsy and forceps biopsy

Author	Depth of tissue	Number of patients in CB, n (%)	Number of patients in FB, n (%)
Baess et al	Fat cells negative	12/24 (50)	8/24 (33.3)
	Fat cells <5%	4/24 (16.7)	8/24 (33.3)
	Fat cells (5-10%)	5/24 (20.8)	2/24 (8.3)
	Fat cells (10-25%)	3/24 (12.5)	6/24 (25)
Dooria et al	Up to mesothelial connective tissue	16/46 (34.8)	29/49 (59.2)
	Up to extrapleural fat or deeper	30/46 (65.2)	20/49 (40.8)
Matura et al	Subpleural adipose tissue	4/6 (66.7)	0/4 (0)
Muhhamad et al	Biopsies containing fatty tissue	21/30 (70)	12/30 (40)
	Biopsies containing no fat tissue	9/30 (30)	18/30 (60)
Nakia et al	Pleural surface	1/5(20)	4/5 (80)
	Adipose tissue	2/5 (40)	1/5(20)
	Skeletal muscle	2/5 (40)	0/5 (0)
Thomas et al	Fibrotic pleura	22/22 (100)	21/22 (95.5)
	Adipose tissue	14/22 (63.6)	5/22 (22.7)
	Skeletal muscle	10/22 (45.5)	2/22 (9.1)
	Lung	1/22 (4.5)	1/22 (4.5)
	Fragments	0/22 (0)	1/22 (4.5)
Wurps et al	Biopsies including fatty tissue	49/99 (49.5)	41/104 (39.4)
	Biopsies Negative for fatty tissue	50/99 (50.5)	63/104 (60.6)

CB, cryobiopsy; FB, Forceps biopsy
